# The single surgeon learning curve of laparoscopic liver resection: A continuous evolving process through stepwise difficulties

**DOI:** 10.1097/MD.0000000000005138

**Published:** 2016-10-28

**Authors:** Federico Tomassini, Vincenzo Scuderi, Roos Colman, Marco Vivarelli, Roberto Montalti, Roberto Ivan Troisi

**Affiliations:** a Department of General, Hepatobiliary and Liver Transplantation Surgery, Ghent University Hospital Medical School; b Bio Cell Statistics, Faculty of Ghent University, Belgium; c Hepatobiliary and Abdominal Transplantation Surgery, Department of Experimental and Clinical Medicine, Polytechnic University of Marche, Ancona, Italy.

**Keywords:** Conversion rate, Laparoscopic learning curve, Laparoscopic liver resection, Laparoscopic liver surgery, Risk-adjusted CUSUM analysis

## Abstract

The aim of the study was to evaluate the single-surgeon learning curve (SSLC) in laparoscopic liver surgery over an 11-year period with risk-adjusted (RA) cumulative sum control chart analysis.

Laparoscopic liver resection (LLR) is a challenging and highly demanding procedure. No specific data are available for defining the feasibility and reproducibility of the SSLC regarding a consistent and consecutive caseload volume over a specified time period.

A total of 319 LLR performed by a single surgeon between June 2003 and May 2014 were retrospectively analyzed. A difficulty scale (DS) ranging from 1 to 10 was created to rate the technical difficulty of each LLR. The risk-adjusted cumulative sum control chart (RA-CUSUM) analysis evaluated conversion rate (CR), operative time (OT) and blood loss (BL). Perioperative morbidity and mortality were also analyzed.

The RA-CUSUM analysis of the DS identified 3 different periods: *P*1 (n = 91 cases), with a mean DS of 3.8; *P*2 (cases 92–159), with a mean DS of 5.3; and *P*3 (cases 160–319), with a mean DS of 4.7. *P*2 presented the highest conversion and morbidity rates with a longer OT, whereas *P*3 showed the best results (*P* < 0.001). Fifty cases were needed to achieve a significant decrease in BL. The overall morbidity rate was 13.8%; no perioperative mortality was observed.

According to our analysis, at least 160 cases (*P*3) are needed to complete the SSLC performing safely different types of LLR. A minimum of 50 cases can provide a significant decrease in BL. Based on these findings, a longer learning curve should be anticipated to broaden the indications for LLR.

## 1 Introduction

Laparoscopic liver surgery (LLS) has gained widespread acceptance in the hepato-biliary community over the last decade due to the overall decreased morbidity, length of hospital stay, and pain and more rapid recovery in selected patients, compared with the standard approach. The worldwide experience was recently updated with the *2nd International Consensus Conference on Laparoscopic Liver Surgery*.^[1]^ Accordingly, 5388 resections, of which 1184 were major resections (22%), have been performed and reported by 18 international specialized centers.^[2]^ The most important technical evolutions introduced during the last few years have included the caudal approach for major hepatectomy, the management of bleeding by performing the Pringle maneuver and temporarily increasing the CO_2_ pressure, the feasibility of anatomical resections and fully laparoscopic living donor hepatectomy.^[^2–7^]^ However, the worldwide spread of laparoscopic liver resection (LLR) is still hindered for the following reasons: the fear of hemorrhage due to underlying liver disease, difficulties in localizing focal liver lesions by ultrasonography and maintaining adequate surgical margins for oncological resections, and the overall lower cumulative incidence of surgical liver pathology compared with that of the bowel.^[^8 9^]^ Achievement of the learning curve (LC) for LLR is therefore longer and demanding, compared with the latter.^[10]^ Many would agree on the need for formal training opportunities during surgical residencies and, consequently, on standardized training programs as a part of HPB surgical fellowships. However, formal specific laparoscopic liver training programs are uncommon. To date, only a few papers have focused on this issue, mainly examining conversion rates (CRs) and blood loss.^[^11–17^]^ However, it is difficult to draw final conclusions because many factors influence LC outcomes, such as the center and team experience, pathology referral, and individual surgical skills, allowing for the reproducibility of open techniques with the laparoscopic approach. Accordingly, the LC for laparoscopic minor hepatectomy is believed to be roughly completed after 60 procedures, allowing for the best performances in terms of conversion rates.^[15]^

LLR was introduced in our institution in 1997, and it was initially limited to resections of small peripheral benign lesions in the anterolateral sectors.^[18]^ Subsequently, a routine program was established with the progressive development of laparoscopic techniques, which are now considered for at least 60% of all liver resections.^[19]^

Considering the lack of data on single surgeon learning curves (SSLC), depicting the ability to evolve through a consistent, consecutive caseload number with stepwise difficulties, we retrospectively evaluated an 11-year period of activity in our institution using the risk adjusted (RA)-CUSUM methodology.^[20]^ Different perioperative variables as well as the types of procedures performed (minor vs major; anatomic vs non-anatomic resections) were considered. The results of SSLC are herein reported.

## 2 Materials and methods

A total of 319 (70.8%) of 450 consecutive laparoscopic liver procedures performed between June 2003 and May 2014 by a single surgeon were retrieved from a prospectively maintained database. Wedge resections, anatomical and non-anatomical segmentectomies, and minor and major hepatectomies for benign and malignant liver diseases were considered in this analysis. Laparoscopic cyst fenestrations, ablation procedures, and combined extra-hepatic surgeries were excluded. Twenty-two fully laparoscopic donor hepatectomies were not included in the statistical analysis due to their rarity and complexity and their exceptional performance, usually requiring longer OT. Neither hybrid nor hand-assisted procedures were included in this series.

Demographic data, indications for LLR, and operative and post-operative data were recorded and retrospectively analyzed. To grade the technical difficulty, a utility score ranging from 1 to 10, expressing the difficulty grade, was assigned to each type of resection, according to the segmental liver anatomy (Table 1).

**Table 1:**
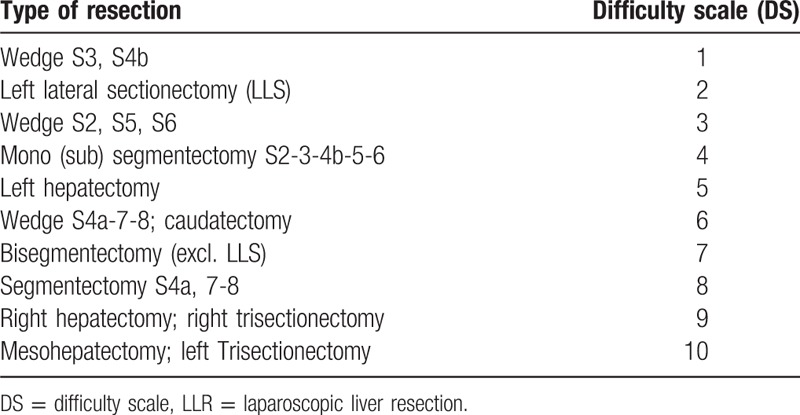
Grading the difficulty: the Difficulty Scale (DS).

This difficulty scale (DS) was empirically developed after discussions and final agreement of 4 European experts in laparoscopic HPB surgery (see Acknowledgments). Resections of 3 or more liver segments were considered to be a major hepatectomy; wedge resections for anterior segments 3 and 4b were classified as the easiest procedures, whereas central hepatectomy was scored with the highest degree of difficulty (Table 1). The selection of the laparoscopic approach was considered each time and was based on the underlying liver disease, the functional remnant for major hepatectomies, and the possibility of obtaining a parenchyma-preserving resection; in all of the cases, all of the LLRs were performed with curative intent.

### 2.1 Surgical technique

The surgical technique has been described elsewhere.^[19]^ The standard technique used in major hepatectomy was upfront unilateral inflow occlusion, followed by upward dissection (caudal to cephalad) from the inferior vena cava. A hanging maneuver with the goldfinger dissector was applied.^[21]^ Intermittent inflow occlusion (Pringle maneuver) was used when necessary (when oozing from fragile parenchyma, failure to control bleeding, or resections of postero-superior segments occurred). Blood loss was calculated by considering the fluid balance and decreases in hemoglobin levels.^[22]^

### 2.2 Evaluation of the learning curve

First, all of the performed procedures were ordered chronologically from the earliest to the latest date of surgery. Cumulative sum control chart (CUSUM) analysis was then applied to the DS to evaluate the progression and evolution of the learning curve over time. The CUSUM of the DS of the first case was the difference between its DS and the mean DS of the entire series. The second case was calculated with the same method and added.^[^20 23^]^

The learning curve (improvement in surgical performance over time) was thereafter defined by the following 3 parameters: conversion rate (CR), blood loss (BL), and operative time (OT). Considering the DS, a risk adjusted-cumulative sum control chart (RA-CUSUM) analysis was performed for the conversion rate. The RA-CUSUM plot considers the predicted probability of conversion according to the DS of each procedure.^[23]^ In this plot, every decrease in the curve represents a conversion, and its depth is related to the DS; an easier procedure conversion is represented by a deeper decrease in the plot. For a case in which conversion occurs, the plot descends as 1 minus the predicted probability of conversion. For a case in which no conversion occurs, the plot increases with the predicted probability of conversion. RA-CUSUM analysis was also applied to evaluate blood loss. In this analysis, for each procedure, the variation of the plot was the difference between the estimated BL and the expected value, calculated using a linear regression in which DS was the predictor variable. The RA-CUSUM of the BL of the first case was the difference between the observed BL and the expected BL for that case. An increase in the plot represented a higher BL than expected.^[24]^

For the operative time, we applied simple CUSUM: the mean surgical time was extracted from each time record to create the plot.

Perioperative morbidity and mortality were considered for events within the first 90 postoperative days. The Dindo–Clavien classification^[25]^ (DC) was used to define specific complications. Major complications were defined by DC classifications of ≥3. Our local IRB approved this study (EC/2016/0076).

### 2.3 Statistical analysis

Patient characteristics are expressed as the mean ± standard deviation for parametric continuous data and as median (IQR) for non parametric. Categorical data are expressed as numbers with percentages. Fisher's exact test was used to compare the differences in categorical variables; ANOVA was used to compare differences in continuous parametric variables when appropriate. The Kruskal–Wallis test was used to compare differences in continuous non-parametric variables when appropriate. Post-hoc analysis was performed using Bonferroni's test in cases of significant differences observed among the 3 groups. Multivariate analyses were performed through a stepwise logistic regression model using conversion as the dependent variable and including significant predictive factors in the univariate analysis. A p-value < 0.05 was considered statistically significant. Statistical analysis was performed using SPSS software (version 20.0. Armonk, NY, IBM Corp) for MacOsX.

## 3 Results

The mean age of the selected patient population was 54.8 ± 16.2 years old, with an F/M ratio of 168/151 (52.7% vs 47.3%). The indications for LLR were benign disease in 119 patients (37.3%) and malignancy in 200 patients (62.7%). The overall morbidity rate was 13.8% (44/319), which is more precisely described as follows: n = 23 for grade I (6.7%), n = 9 for grade II (2.6%), n = 11 for grade III (3.2%), and n = 1 for grade IV (0.3%) (Table 2). Precisely, the grade III complications included the following: biliary leakage in 3 (0.9%) cases, pneumothorax in 4 cases (1.2%), intra-abdominal abscess in 2 cases (0.6%), and intra-abdominal bleeding and pleural effusion in 1 (.3%) case each. One patient presented a lung embolism defined as grade IV. No 90 day perioperative mortality was recorded.

**Table 2:**
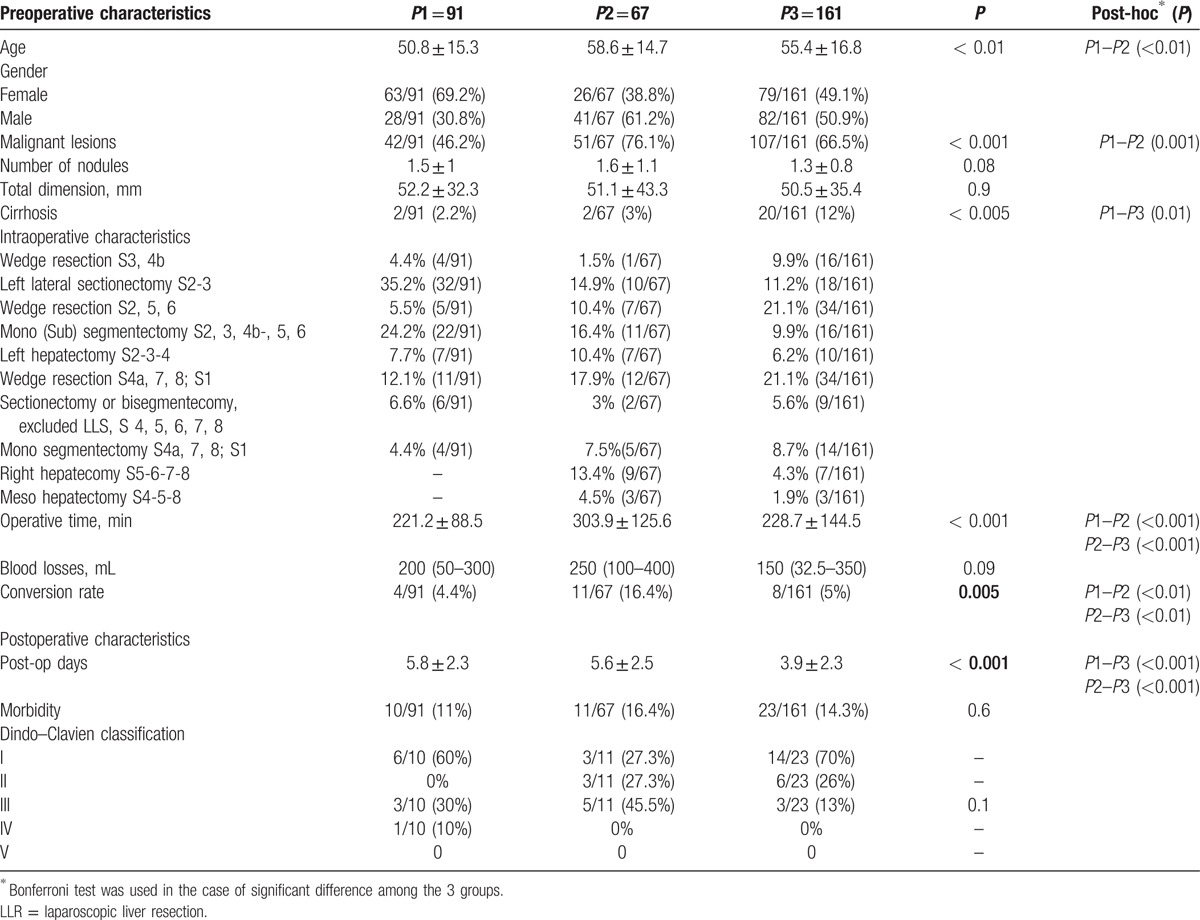
Patients characteristics and perioperative data.

### 3.1 Stepwise difficulty

**Figure 1:**
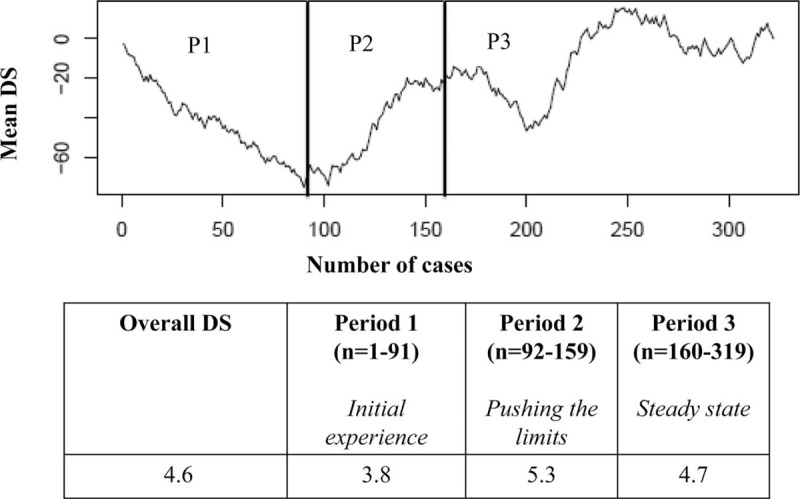
CUSUM analysis of the DS. DS = difficulty scale, CUSUM = cumulative sum control chart.

According to the CUSUM analysis of the DS, the following 3 periods were identified: period 1 (*P*1), termed “*the initial experience,”* n = 91 cases; period 2 (*P*2), termed *“pushing the limits”,* from case 92 to case 159, n = 67 cases; and period 3 (*P*3), termed *“the steady state”,* from case 160 to 319 (n = 161). The average DS was 3.8 (the lowest), 5.3 (the highest) and 4.7 (the steady state), respectively, for periods 1, 2, and 3 (*P* < 0.001) (Fig. 1). The preoperative and intraoperative characteristics among the 3 different periods are summarized in Table 2. Minor resections and/or non-anatomical resections were predominantly performed during *P*1. The first left hepatectomy was performed after 17 cases with the highest incidence of major hepatectomies during *P*2 (28.4% vs 7.7% and 11.2% in *P*1 and *P*3, respectively, *P* < 0.001). During *P*2, the postero-superior segments as well as right hepatectomies were progressively approached. Resections in cirrhotic patients were globally very limited, with the significantly highest rate in *P*3 (2.2%, 2.9% and 12%, respectively, for *P*1, *P*2, and *P*3, *P* < 0.005). The intermittent Pringle maneuver was performed in 48 cases (15%), predominantly during *P*3 (22.4% vs 6.6% and 9%, respectively, for *P*1 and *P*2; *P* = 0.001).

### 3.2 Indications

During the study period, there was an increase in LLR for primary and secondary malignant lesions. During *P*1, liver resections for malignancies were performed in 46.1% of the cases, compared with 76.5% and 66.5% in *P*2 and *P*3, respectively (*P* < 0.001, Table 2).

### 3.3 Conversion rate

**Figure 2:**
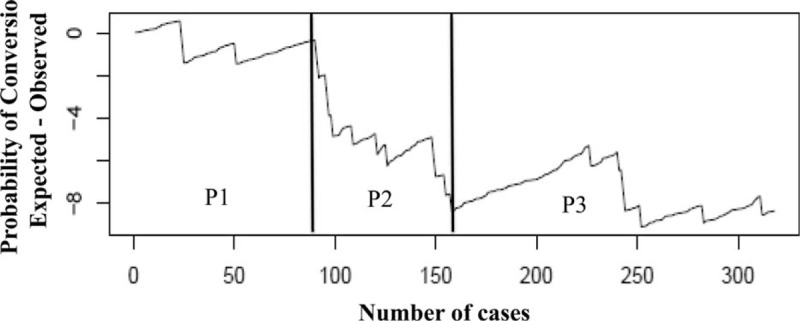
RA-CUSUM analysis of conversion rate. RA-CUSUM = risk-adjusted cumulative sum control chart.

The overall conversion rate was 7.2% (23/319), with the highest rate during *P*2 (16.4%, *P* = 0.005, Table 2). The RA-CUSUM plot of the conversion rate confirmed and showed the highest risk of conversion during *P*2, during which more conversions than expected were observed. After 160 procedures, we observed a better trend, with the lowest conversion rate attained between 200 and 250 procedures (Fig. 2). Univariate analysis showed that total tumor dimension (>50 mm), major hepatectomy, resection of lesions located in postero-superior segments, blood loss (>500 mL) and the need for intermittent Pringle maneuver were independent variables predicting conversion. In multivariate analysis, major hepatectomy was the only confirmed predictive factor for conversion (*P* = 0.002, OR = 21.1, 95% CI 3.1–142.3) (Table 3). The most common reasons for conversion were the presence of adhesions and bleeding in 7 (30.4%) cases each. Five other patients (21.7%) were converted for oncological reasons, 3 due to CO_2_ embolisms, and 1 for anatomical reasons (unclear intrahepatic anatomy with portal vein trifurcation).

**Table 3:**
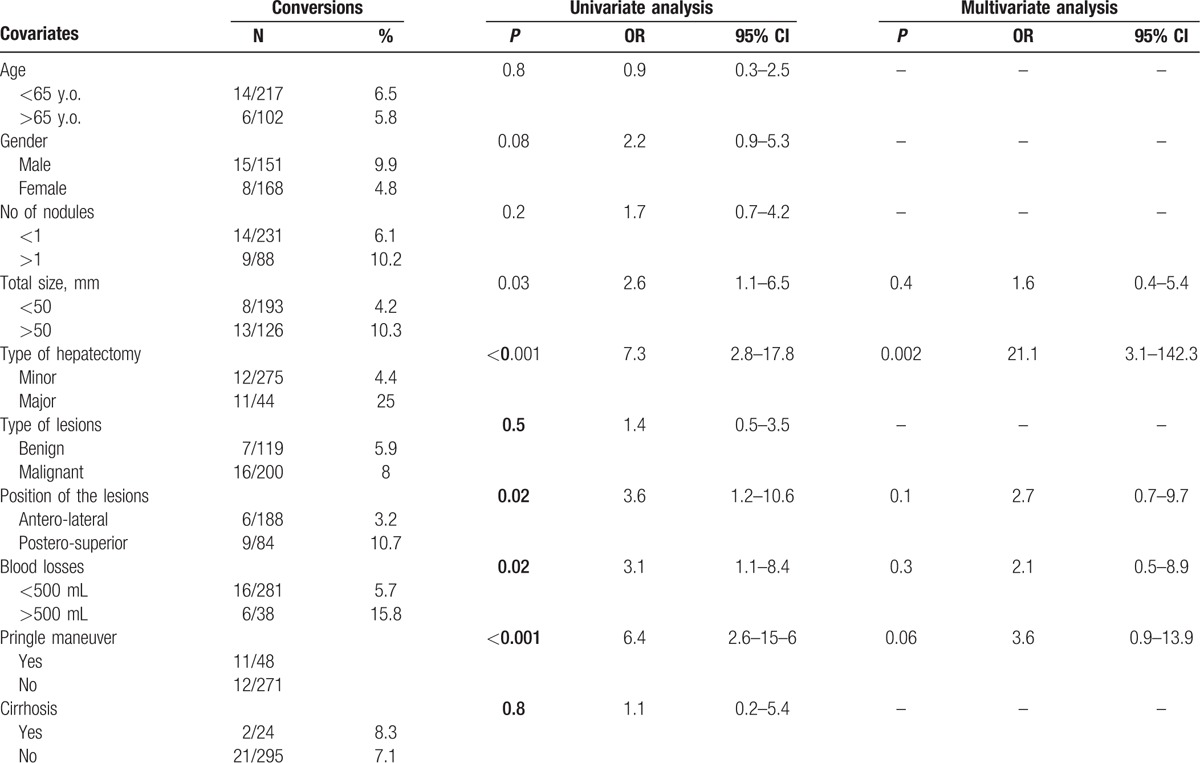
Logistic regression analysis of the predictive variables of conversions.

### 3.4 Operative time

**Figure 3:**
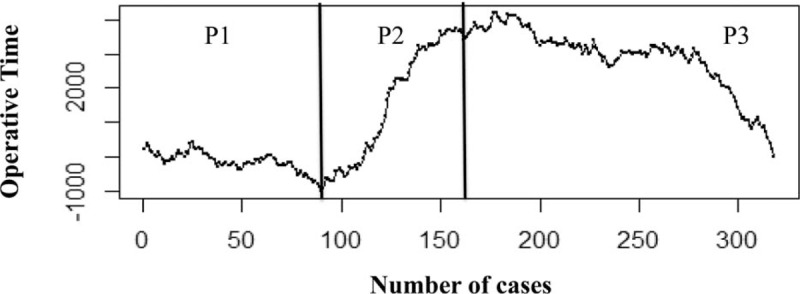
CUSUM analysis of operative time. CUSUM = cumulative sum control chart.

The mean OT over the whole series was 231.7 ± 114 min, and the statistically significantly longest OT was recorded during *P*2 (303.9 ± 125.6, *P* < 0.001, Table 2). The CUSUM analysis confirmed and clearly showed that the longest OT was recorded in *P*2, and the best performances were obtained in *P*3 (Fig. 3).

### 3.5 Blood loss

**Figure 4:**
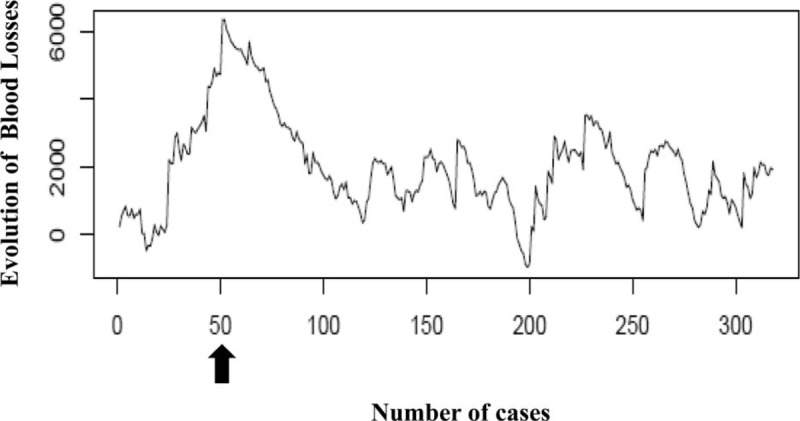
RA-CUSUM analysis of blood losses. RA-CUSUM analysis of blood losses. The arrow indicates the best results after 50 LLR. LLR = Laparoscopic liver resection, RA-CUSUM = risk-adjusted cumulative sum control chart.

The overall median BL was 200 (50–400) mL, and no differences were recorded among the 3 periods (Table 2). The RA-CUSUM plot showed an upward trend during the first 50 cases. From case 51 onward, a progressive decrease in BL was recorded, with the best results after approximately 200 LLR cases (Fig. 4).

### 3.6 Surgical morbidity

The highest morbidity rate was recorded during *P*2 (16.4% vs 11% and 14.3% in *P*1 and *P*3, respectively, *P* = 0.6). Moreover, in *P*2, we observed the highest percentage of grade III complications (30%, 45.5%, and 13%, respectively, for *P*1, *P*2, and *P*3 – *P* = 0.1). Only 1 patient presented a grade IV complication during *P*1 (lung embolism, medically treated). During *P*3, we observed the shortest mean length of hospital stay (3.9 ± 2.3 days, *P* < 0.001) (Table 2).

## 4 Discussion

The *2* ^*nd*^ *International Consensus Conference* on Laparoscopic Liver Surgery confirmed the increasing popularity of laparoscopic liver surgery over the previous 6 years following the Louisville statement.^[^1 26^]^ However, no suggestions could be provided regarding how to provide the best teaching and training that would eventually shorten the learning curve. The absolute need for a well-defined educational process to facilitate the diffusion of laparoscopic techniques was acknowledged.^[1]^ With perfect knowledge of liver anatomy and advanced skills in laparoscopy and liver surgery, trainees in expert centers can begin performing laparoscopic liver resections. Traditional teaching programs include video discussions, courses on cadavers and laparoscopic surgery in animal models, but after formal teaching, the real single surgeon learning curve remains unclear, and the learning process remains lengthy due to the difficulties of its reproducibility. Different aspects have been analyzed and related to laparoscopic LC, mainly focusing on 1 specific procedure or single outcome. Vigano et al^[15]^ described, for the first time, LC based almost exclusively on a CUSUM analysis of conversions and a comparison of 3 different time periods. The authors concluded that LLR was reproducible in specialized HPB units, and 60 procedures constituted the cut-off point to attain a minimal conversion rate. However, 4 different surgeons shared this experience. Moreover, Cai et al^[13]^ described a single center experience regarding the LC in LLR for 4 different well-defined procedures, claiming 15–30 cases of left hemihepatectomy, 43 cases of left lateral sectionectomy, 35 cases of non-anatomic liver resection and 28 segmentectomies as the numbers of procedures needed to achieve the best perioperative results.

Nomi et al^[17]^ recently published a paper focusing on the LC for laparoscopic major hepatectomies. Using the CUSUM analysis of the OT to define 3 different periods, they suggested that 45 LMH procedures were required to reduce the OT and to move from phase 1 to phase 2.

Because the published evidence has focused on a single center experience, often represented by different surgeons and/or based on few procedures, we attempted to analyze the single surgeon learning curve as a continuously evolving process through stepwise difficulties by considering 11 years of experience.

However, a detailed analysis cannot determine the degree of difficulty. Currently, there is no common opinion about the degree of difficulty of LLR. Ban et al^[27]^ attempted to develop a mathematic difficulty scoring system by combining the opinions of different expert surgeons in Japan. Because our CUSUM analysis of the surgical outcomes was unable to focus on the stepwise evolution of the SSLC, we decided to structure the RA-CUSUM statistical analysis using an empirical scale to express the degree of difficulty according to the experience of 4 experts in LLS. This DS ranged from 1 to 10, with a difficulty grade assigned to every single procedure performed according to the segmental anatomy and the type of resection. Consequently, the SSLC was described, focusing on 4 variables (conversions, operative time, blood loss, and surgical morbidity rate) that are believed to be indirect signs of the learning curve. During the study period, we observed an increased trend toward approaching malignancies over time and a stable trend in minor hepatectomies, reflecting our propensity for the parenchyma-saving approach, which is indeed the standard of care for the vast majority of liver lesions, except for HCC.^[^22 28^]^

Our results showed 3 different periods according to the trend in the DS, as follows: *P*1, which can be considered the initial experience; *P*2, which can be considered the period in which the surgeon attempted to push his limits because of gained confidence and expertise, trying to address the most challenging procedures; and finally, *P*3, which should be considered the completion of the LC. The highest conversion rate was found during *P*2, with a better trend between 200 and 250 procedures performed (*P*3). Moreover, during *P*3, we observed the lowest cumulative conversion rate. The total dimensions of lesions, major hepatectomy, resection of lesions located in postero-superior segments, blood loss and the need for the intermittent Pringle maneuver were variables predicting conversion. However, as reported by others, major hepatectomy was the only independent factor predictive of conversion in multivariate analysis.^[17]^

Considering the Dindo-Clavien grade III and IV complications, the best results were also attained during *P*3, showing that the morbidity rates were inversely correlated with the technical proficiency. The decreased morbidity and the absence of 3-month perioperative mortality over the whole series demonstrated the safety and feasibility of the laparoscopic technique in expert hands.

The analysis of the operative time showed the longest value during *P*2 and the best results in *P*3. Most likely, this finding was related to the difficulty of the procedures, in which the surgeon engaged himself after the initial part of his activity. Accordingly, to master different types of liver procedures, a minimum of 160 LLRs (corresponding to the end of *P*2) should be considered to complete the learning curve. Some might believe this volume to be excessive and too daunting; however, we note that all types of procedures were included in our analysis, even the most difficult (i.e., postero-superior segments), for which the role of the laparoscopic approach is still under debate.

Our results confirmed 2 important aspects. First, in high volume centers, with large, consecutive LLR series, it is possible to obtain progressively complete laparoscopic proficiency without risking patient safety. Second, the LC of LLR is definitely long and challenging, requiring constant and progressive commitment, which also foreshadows the role of teaching young fellows. As explicitly required by the second World Conference, major efforts are required to determine how the laparoscopic skills needed for difficult procedures should be obtained by trainees and HPB surgeons in practice.^[1]^

In contrast, the analysis of the BL showed different results that were not comparable with the other outcomes analyzed and did not match the 3 chosen periods. Accordingly, after 50 LLRs, we recorded a decreased trend in blood loss, with the best results attained after approximately 200 procedures. Considering this result, we could conclude that, after 50 cases, a laparoscopic surgeon can already manage the techniques, applying some useful tricks to reduce blood loss during parenchymal transection. This finding could be explained as the concrete understanding of the advantages and limitations of available instruments and the mastery of the fundamental techniques of bleeding control in LLR, leading to the transition between *P*1 and *P*2. In fact, in our institution, parenchymal transection is performed with the ultrasonic surgical aspirator, whereas bipolar forceps are used to refine the hemostasis on the cutting edge. The Pringle maneuver is applied in selected cases (i.e., postero-superior segments, cirrhosis, chemotoxicity), whereas graspers, clips, sutures, and linear staplers are selectively used to assure hemostasis or to stop the bleeding from intrahepatic vessels.

This study had different limitations. First, the reproducibility of an SSLC was related to the single surgeon's skills, and his training could not be clearly defined and evaluated in this statistical analysis. Second, our DS is a subjective evaluation of the difficulty of a single LLR, not considering other variables, such as patient characteristics (BMI or ASA score), the quality of the liver and the relationship of the tumoral mass with the intrahepatic anatomy.

In conclusion, according to our analysis, the SSLC could be considered completed after 160 cases, in which the surgeon progressively challenged himself with various procedures through stepwise difficulties; the gradual increase in the types of procedures and degree of difficulty has led successively to the safe management of major hepatectomies and resections of postero-superior segments. Based on these findings, a long learning curve should be anticipated to broaden the indications for LLR. We believe that an established LLS program could be conceived only in centers with sufficient expertise in HPB surgery, including basic laparoscopic knowledge of the abdominal pathology. Certainly, in laparoscopic liver surgery, the continuous monitoring of performance and results is a crucial step in completing the LCs of younger fellows, who will master laparoscopic techniques in the future.

## Acknowledgments

The authors wish to thank Bjorn Edwin (Oslo), Mohammed Abu-Hilal (Southampton), and Luca Aldrighetti (Milan) for their help in defining the difficulty scale described in this manuscript.
